# *Rattus norvegicus* and *Rattus tanezumi* as hub hosts for viral diversity and potential spillover on Hainan Island, China

**DOI:** 10.1038/s41522-026-01056-x

**Published:** 2026-06-22

**Authors:** Haoyue Huangfu, Ji Pu, Mengqi Jiao, Qianjin Fan, Huiwei Deng, Qian Ling, Hao Zhou, Xuelian Luo, Jianguo Xu

**Affiliations:** 1 School of Medicine, Nankai University, Tianjin, PR China; 2 National Key Laboratory of Intelligent Tracking and Forecasting for Infectious Diseases, National Institute for Communicable Disease Control and Prevention, Chinese Center for Disease Control and Prevention, Beijing, PR China; 3 School of Public Health, Peking University, Beijing, PR China; 4 Research Center for Reverse Microbial Etiology, Workstation of Academician, Shanxi Medical University, Taiyuan, PR China

**Keywords:** Ecology, Ecology, Microbiology, Zoology

## Abstract

Rodents, which account for over 40% of mammalian species and occupy diverse terrestrial ecosystems, are major reservoirs for zoonotic viruses, including *Coronaviridae*, *Hantaviridae*, and *Arenaviridae*. However, the rodent species acting as central hubs for viral diversity and transmission remain poorly defined. To address this, we conducted systematic sampling across 18 counties and cities on Hainan Island between 2023 and 2024. A total of 2550 animals were collected, representing 14 Rodentia species, one Erinaceomorpha species, and one Scandentian species. Based on a statistical sampling framework, 1284 individuals were selected to generate 125 pooled gut samples for virome analysis. We identified 527 viral RNA species and recovered 175 complete or near-complete viral genomes. Remarkably, *Rattus norvegicus* and *Rattus tanezumi* accounted for 83.9% (442/527) of detected viral species, 68.6% (72/105) of novel viral sequences, and 88.6% (93/105) of cross-species viruses. Eight novel viruses showed recombination signals, seven originating from these two species. Among 13 human pathogenic viruses identified, 12 were detected in *Rattus norvegicus* and *Rattus tanezumi*. These findings suggest that these species may function as hub hosts for viral maintenance and spread, emphasizing the value of targeted surveillance to reduce future potential spillover risks.

## Introduction

Approximately 75% of emerging infectious diseases (EIDs) originate from animals, underscoring the critical connection between wildlife and human health^1^. This risk is particularly acute for rodents since viral diversity often increases with host diversity^2^, and the order Rodentia constitutes the most diverse mammalian group^3^, with over 2200 species acting as reservoirs for many zoonotic pathogens^3–5^. High-throughput sequencing has revealed the enormity of this threat by identifying a wide range of relevant zoonotic viruses, including members of the *Hantaviridae, Arenaviridae*^6,7^, and *Coronaviridae* families^8^. A major obstacle to mitigating this threat, however, is that the key reservoir species for these viruses remain largely unknown, creating a serious vulnerability in our pandemic preparedness.

Hainan Island is a recognized biodiversity hotspot in southern China where the tropical climate supports dense rodent populations^3^. Concurrently, rapid urbanization and agricultural expansion are increasingly bridging natural habitats with human communities, fostering ideal conditions for pathogen spillover^3^. Despite these converging risk factors, comprehensive virome studies targeting the island’s rodent populations remain scarce. This data gap substantially limits our understanding of local viral diversity and associated zoonotic risks, thereby undermining proactive health security measures in a region that also serves as a major international hub for trade and tourism.

To address this critical viromic data gap and identify key hosts involved in potential cross-species viral transmission, we conducted a meta-transcriptomic virome survey of 16 small mammal species—comprising 14 Rodentia species, one Erinaceomorpha species, and one Scandentian species—across 18 cities and counties in Hainan Island (excluding Sansha). Our objectives were to characterize viral communities across host species and sampling regions (northern vs. southern Hainan), to identify novel viruses and those with cross-species transmission potential, and ultimately to establish a foundational viromic dataset for the province. By doing so, this study provides a scientific basis for developing targeted surveillance frameworks and early-warning strategies—essential components in the effort to mitigate future zoonotic spillover and enhance public health preparedness.

## Results

### The virome of small mammals

**Fig. 1:**
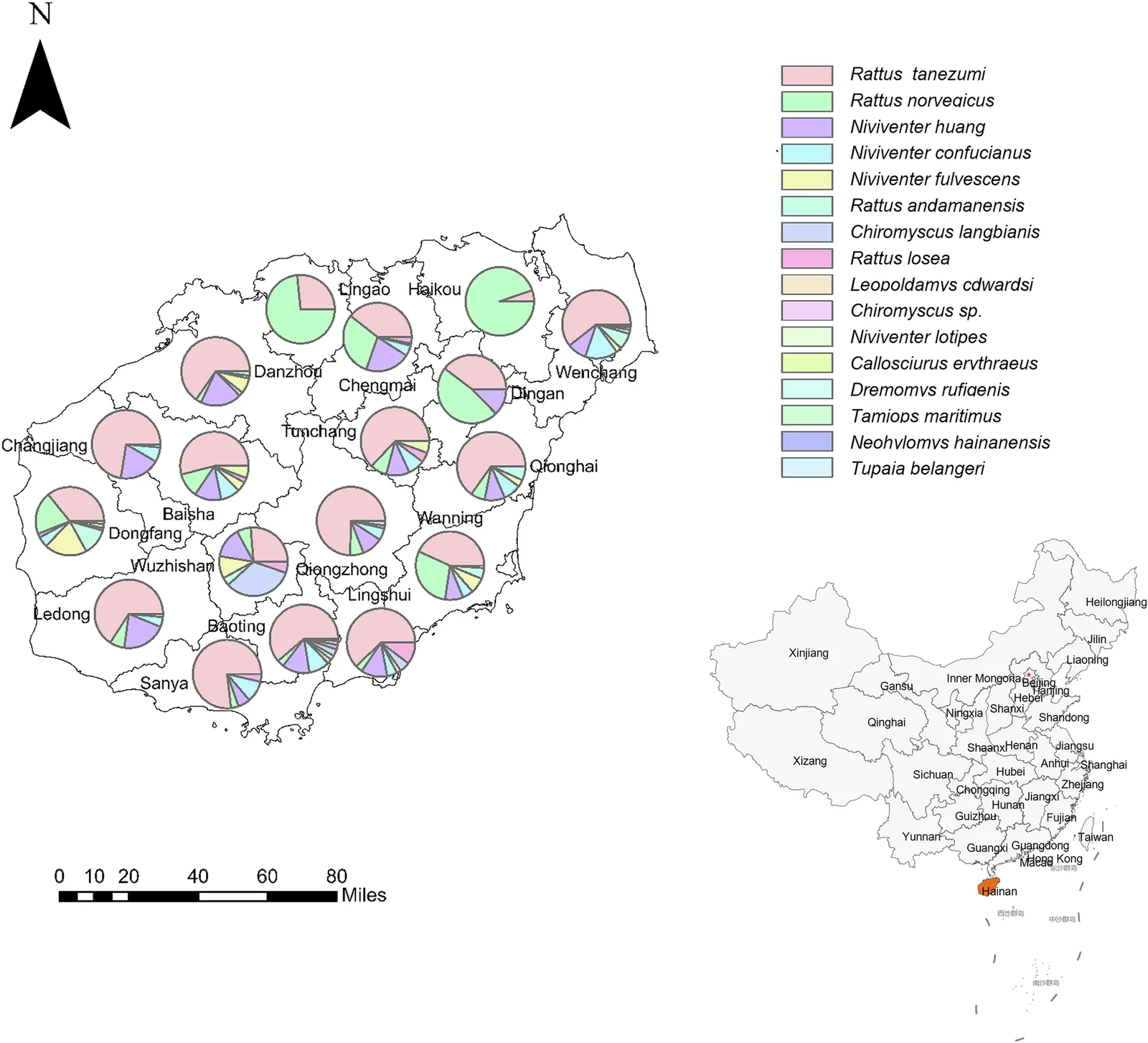
Sample distribution of 16 small mammal species in this study across counties/cities in Hainan Province, China (excluding Sansha). A total of 2550 individual animals were collected between June 2023 and December 2024. Pie charts display sample composition by species (color-coded) and collection count per site (white). The distribution map was generated using ArcMap 10.8 software.

To establish a virome profile of small mammals on Hainan Island, China, we sampled 2550 individuals—including 14 rodent species, one *Neohylomys hainanensis* (Erinaceomorpha), and one *Tupaia belangeri* (Scandentia) (Supplementary Fig. 1)—across 18 cities and counties throughout the island (excluding Sansha) between June 2023 and December 2024. Sampling was conducted across a broad range, with sample sizes per location as follows: Dongfang (*n* = 161), Wanning (*n* = 178), Wenchang (*n* = 155), Qionghai (*n* = 180), Baoting (*n* = 277), Wuzhishan (*n* = 76), Chengmai (*n* = 100), Ding’an (*n* = 63), Tunchang (*n* = 129), Danzhou (*n* = 151), Changjiang (*n* = 133), Baisha (*n* = 87), Lingao (*n* = 206), Sanya (*n* = 139), Lingshui (*n* = 145), Ledong (*n* = 130), Qiongzhong (*n* = 145), and Haikou (*n* = 95) (Fig. 1 and Supplementary Table 1).

Based on a stratified sampling strategy that considered both sampling locations and host species, we constructed 125 pools for subsequent metatranscriptomic sequencing. Total RNA sequencing generated approximately 36.7 billion high-quality paired-end reads (150 bp each). Following rigorous quality control, adapter trimming, and error correction, these reads were assembled into 7198 viral contigs, collectively representing 134,985,955 viral-derived reads. Through BLASTx alignment analysis^9^, viral contigs were assigned to 45 recognized RNA viral families within the realm *Riboviria*, while a subset of contigs could not be confidently classified at the family or order level and were therefore designated as unclassified RNA viruses. Meanwhile, 5341 contigs associated with bacterial, fungal, or plant viruses were excluded from further analysis.

**Fig. 2:**
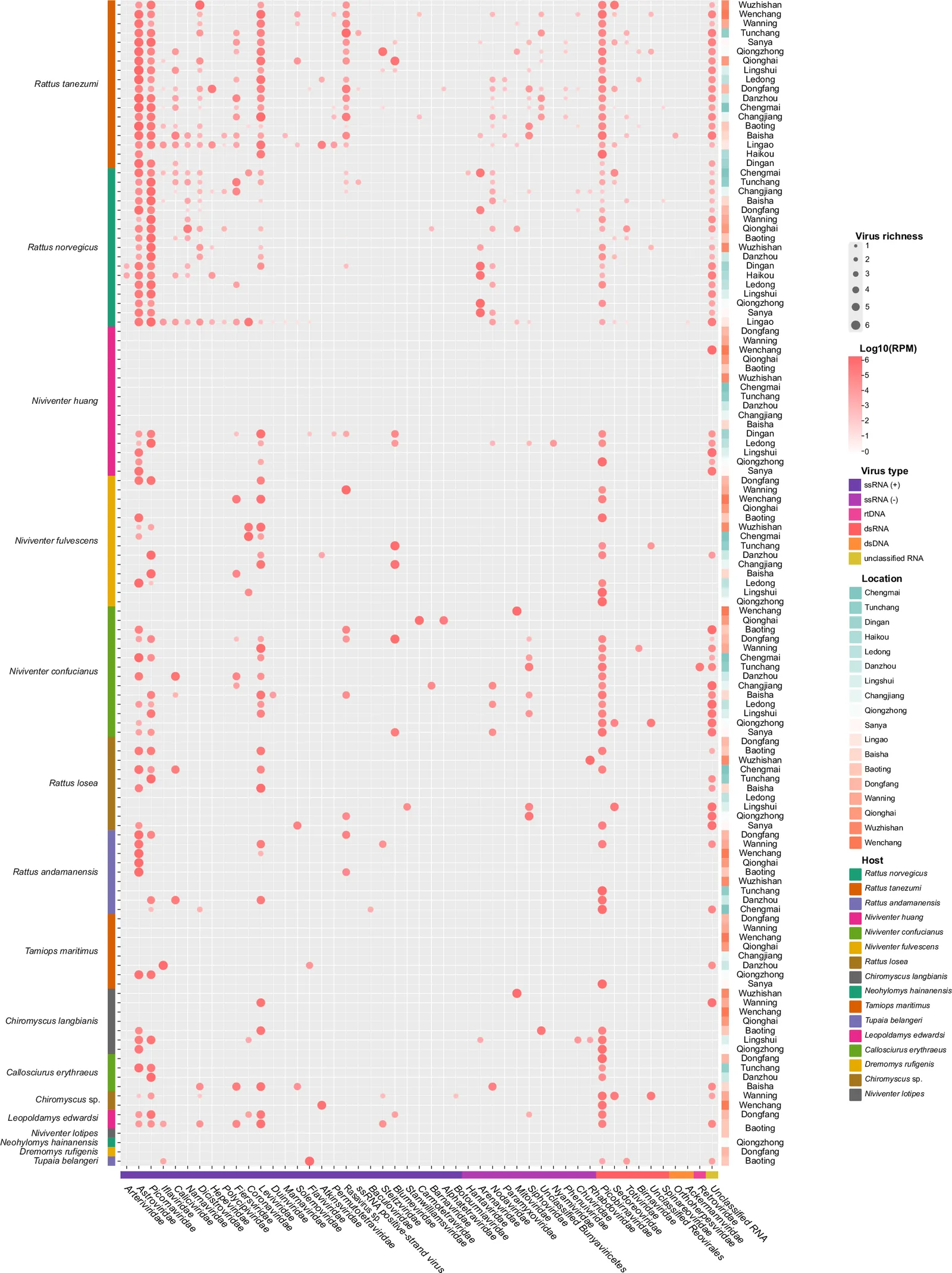
An overview of the virome of 16 small mammal species in this study. Species richness and viral abundance in 16 small mammal species in this study were assessed. Viruses identified in this study all belong to the realm *Riboviria* and were classified into 45 RNA viral families where possible. Viral genomes were further grouped by genome organization into positive-sense ssRNA, negative-sense ssRNA, dsRNA, and unclassified RNA viruses. The relative abundance of viruses in each library was calculated and normalized based on total reads per million (RPM). In the heatmap, columns represent pooled libraries grouped by host species and sampling counties/cities; rows represent viral families or genome types. Color intensity represents viral abundance normalized by reads per million (RPM).

Based on alignments against the non-redundant (NR) database, we compiled the virome profile of 16 small mammal species from tropical Hainan Island (Fig. 2). Furthermore, the virus family composition varied among the small mammal species, and across two regions of sampling locations where the small mammal species were collected (Supplementary Fig. 2). Among these, we identified several families—including *Astroviridae*, *Flaviviridae*, *Iflaviridae*, *Picobirnaviridae*, *Picornaviridae*, and *Caliciviridae*—that demonstrated persistent circulation across multiple host species, suggesting their established transmission within the ecosystem. Additionally, *Picobirnaviridae* exhibited the highest identification frequency (*n* = 74) across families, followed by *Astroviridae* (*n* = 68) (Supplementary Table 7).

### Viral community analysis

To evaluate viral community variation, we used beta diversity metrics (Adonis test) and alpha diversity indices (observed richness and Shannon index)^10^.

**Fig. 3:**
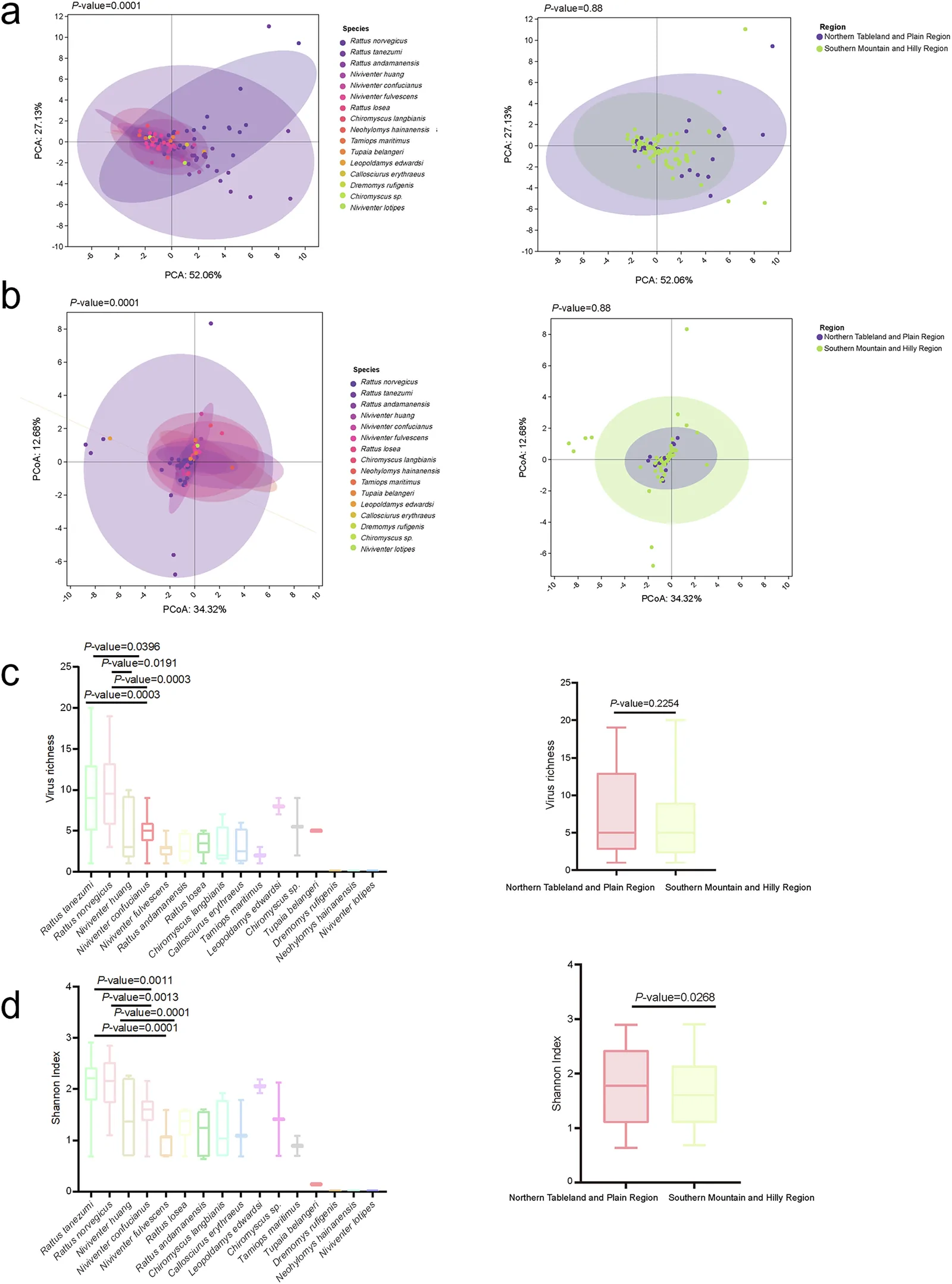
Viral community variation across species and sampling regions in this study. **a** Principal Coordinate Analysis (PCoA) plot: virome variation across 16 small mammal species and two sampling regions (Northern and Southern) in this study. **b** Principal component analysis (PCA): virome variation species and two sampling regions in 16 small mammal species in this study. All *P* values by the Adonis test. **c** Virus richness and **d** Shannon index across 16 small mammal species and two sampling regions in this study. All *P* values by T test.

Beta diversity analysis demonstrated statistically significant differences in virome composition that were associated with species (adonis: *P* = 0.0001; Fig. 3a, b). Alpha diversity analysis revealed significant variations in viral community composition across the 16 species examined, with *Rattus tanezumi* and *Rattus norvegicus* showing particularly distinct patterns. Specifically, both *Rattus tanezumi* and *Rattus norvegicus* exhibited significantly greater virus richness compared to eight other species (*P* < 0.05; Fig. 3c): *Niviventer confucianus*, *Niviventer fulvescens*, *Rattus andamanensis*, *Rattus losea*, *Niviventer huang*, *Chiromyscus langbianis*, *Callosciurus erythraeus*, and *Tamiops maritimus*. Analysis based on the Shannon index further corroborated these findings: *Rattus tanezumi* displayed significantly higher diversity than five other species (*Niviventer confucianus*, *Niviventer fulvescens*, *Rattus losea*, *Rattus andamanensis*, *Chiromyscus langbianis*), while *Rattus norvegicus* showed significantly higher diversity than six other species (*Niviventer confucianus*, *Niviventer fulvescens*, *Rattus losea*, *Rattus andamanensis*, *Chiromyscus langbianis*, *Tamiops maritimus*) (*P* < 0.05; Fig. 3d; Supplementary Table 3). Collectively, these results establish that *Rattus tanezumi* and *Rattus norvegicus* harbor a significantly broader range of viruses than the other species included in this study.

At the geographic level, previous studies have established that Hainan Island exhibits a distinct north-south division characterized by contrasting topographic and ecological features, comprising the Northern Tableland and Plain Region (comprising Lingao, Wenchang, Chengmai, Ding’an, Tunchang, and Haikou) and the Southern Mountain and Hilly Region (comprising Dongfang, Wanning, Qionghai, Baoting, Wuzhishan, Danzhou, Changjiang, Baisha, Qiongzhong, Sanya, Ledong, and Lingshui) as two major geographical units^11,12^. Beta diversity analysis revealed variations in virome composition across these sampling regions (adonis: *P* = 0.88; Fig. 3a, b). Virus richness was observed to be higher in the Northern Tableland and Plain Region than in the Southern Mountain and Hilly Region (*P* = 0.2254; Fig. 3c). Similarly, the Shannon index demonstrated a statistically significant elevation in the Northern Tableland and Plain Region compared to the Southern Mountain and Hilly Region (*P* = 0.0268; Fig. 3d; Supplementary Table 3). In addition to host taxonomy and sampling location, we evaluated the influence of several additional metadata variables—including season, geographic coordinates, altitude, ambient temperature, relative humidity, and collection time—on gut virome composition. None of these factors showed statistically significant associations with viral α-diversity or β-diversity (all *P* > 0.05).

### Phylogenetic analysis of novel RNA viruses

**Fig. 4:**
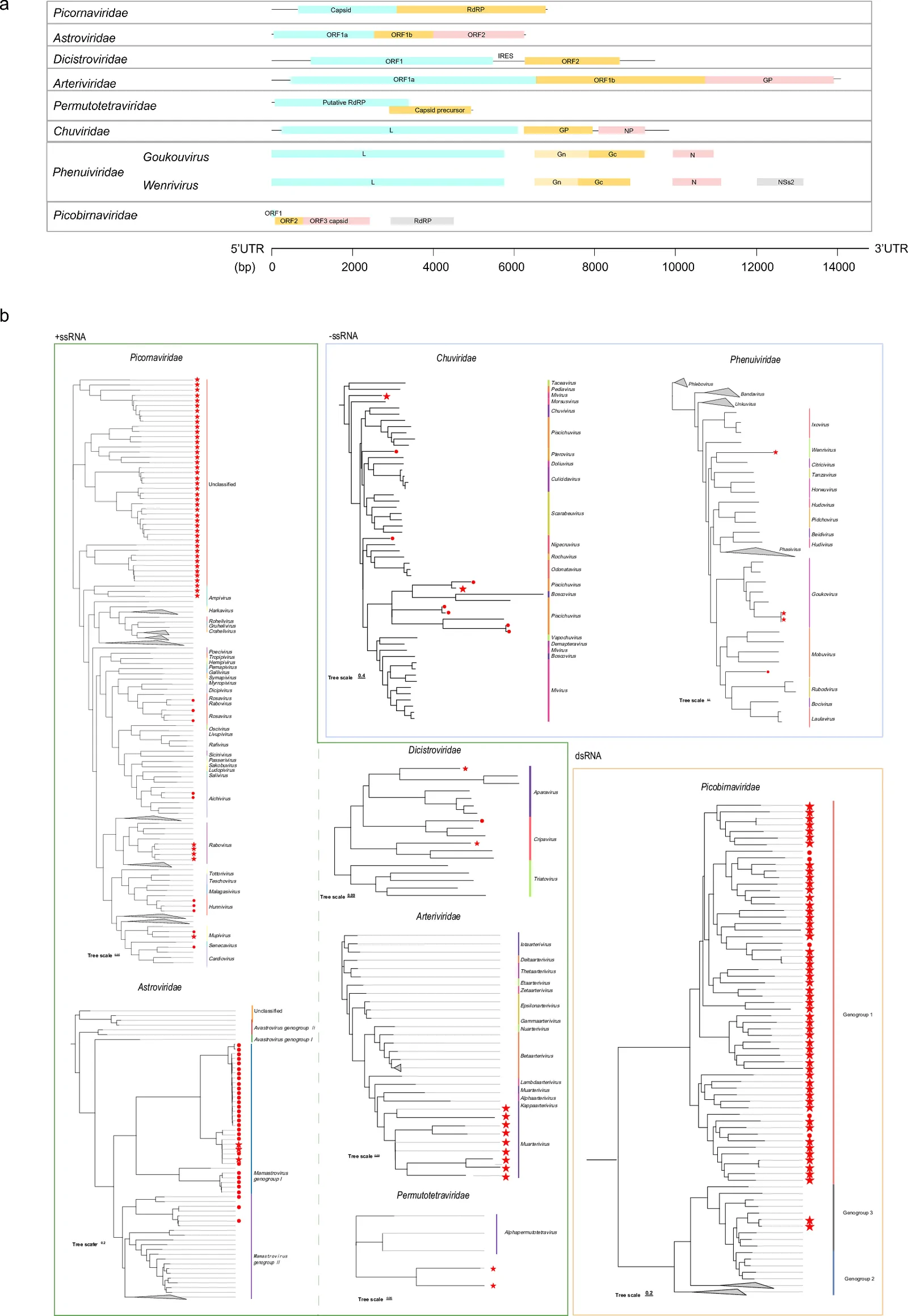
Genomic structure and phylogenetic analysis of novel viruses spanning eight viral families. **a** Genomic architecture of near-complete mammal-associated viral genomes. Viral families/genera are listed on the left. Color-filled rectangles represent Open Reading Frames (ORFs), with distinct colors denoting different ORFs. Different viral families are highlighted with light gray boxes. **b** A phylogenetic tree was constructed based on the aa sequences of the RdRP proteins from the identified novel RNA viruses. Viral families containing *Picornaviridae*, *Astroviridae*, *Dicistroviridae*, *Arteriviridae*, *Permutotetraviridae*, *Chuviridae*, *Phenuiviridae* and *Picobirnaviridae*. The novel viruses identified in this study are marked in red stars. The best-fit substitution model was determined using the ModelFinder program implemented in FastTree (v2.1.11). Phylogenetic inference was performed using the maximum likelihood (ML) method, with branch support evaluated through 1000 bootstrap replicates. Branch lengths are scaled and indicated by a scale bar.

Based on current ICTV13 criteria^13^, we identified 292 novel viruses, spanning positive-sense ssRNA (*n* = 55), negative-sense ssRNA (*n* = 4), dsRNA (*n* = 54), and unclassified RNA viruses (*n* = 179) (Supplementary Table 4). From this collection, we obtained 105 novel viral sequences (Supplementary Table 6; Fig. 4a)—of which 68.6% (72/105) originated from *Rattus tanezumi* and *Rattus norvegicus*—and used them to perform phylogenetic reconstruction based on their RdRP sequences (Fig. 4b). This analysis encompassed eight viral families: *Picobirnaviridae* (54), *Picornaviridae* (30), *Arteriviridae* (9), *Astroviridae* (3), *Phenuiviridae* (3), *Chuviridae* (2), *Dicistroviridae* (2), and *Permutotetraviridae* (2) (Supplementary Table 5).

*Permutotetraviridae* family comprises non-enveloped viruses with a positive-sense, single-stranded RNA genome, consisting of one linear segment^13^. Two novel permutotetraviruses were identified in gut samples from *Rattus tanezumi* collected in Lingao. The first, Hainan rodent permutotetravirus 1, shares 65.04% aa identity with a permutotetravirus isolated from *Miniopterus schreibersii*. The second, Hainan rodent permutotetravirus 2, shows 66.12% identity to an alphapermutotetravirus sp. derived from a bird host. Phylogenetic analysis indicates that both sequences cluster within the *Permutotetraviridae* family, with Hainan rodent permutotetraviruses forming a distinct branch closely related to mammalian-derived alphapermutotetraviruses.

*Dicistroviridae* is a family of small, non-enveloped, positive-sense, single-stranded RNA viruses^14^. Two novel dicistroviruses with near-complete genomes were identified in rodent hosts from Hainan Island. Hainan rodent dicistrovirus 1 was identified in *Rattus norvegicus* from Lingao, showing 77.73% aa identity to *Aparavirus*. Hainan rodent dicistrovirus 2 was identified in *Rattus tanezumi* from Dongfang, exhibiting 77.23% aa identity to Phenacoccus solenopsis virus (from the mealybug *Phenacoccus solenopsis*). Phylogenetic analysis reveals these novel viruses form distinct lineages within the *Dicistroviridae* family, with Hainan rodent dicistrovirus 1 clustering within the *Aparavirus* genus and Hainan rodent dicistrovirus 2 within the *Cripavirus* genus.

*Chuviridae* is a family of negative-sense RNA viruses characterized by genomic diversity, encompassing non-segmented linear and non-segmented circular^15^. Two novel Chuviruses were identified in different rodent hosts from distinct sampling locations. Hainan rodent chuvirus 1 was identified in *Rattus norvegicus* from Changjiang, exhibiting 68.15% aa identity to Lampyris noctiluca chuvirus-like virus 1 (originating from *Lampyris noctiluca*). Hainan rodent chuvirus 2 was identified in *Rattus tanezumi* from Dongfang, showing 74.07% aa identity to Hubei chuvirus-like virus 3 (from Odonata insects). Phylogenetic analysis reveals both two novel rodent-associated chuviruses form distinct lineages within the *Chuviridae* family, demonstrating closer genetic relationships to arthropod-origin chuviruses than to other known rodent-associated strains.

*Phenuiviridae* are enveloped, negative-sense, single-stranded RNA viruses with segmented genomes, typically comprising three segments^16^. Three novel phenuiviruses were identified within *Rattus tanezumi* collected from different sampling locations in this study. Hainan rodent phenuivirus 1, obtained from Qiongzhong, shared 72.28% aa identity with Jingmen bat phenuivirus 1 (derived from *Myotis ricketti*). The other two strains, Hainan rodent phenuivirus 2 and Hainan rodent phenuivirus 3, showed 68.77% and 69.85% aa identity, respectively, with the same reference virus, Hymenopteran phenui-related virus (from *Ceraphron*). Phylogenetic analysis reveals that these novel sequences form distinct clades within the *Phenuiviridae* family.

*Astroviridae* is a family of small, non-enveloped, positive-sense RNA viruses that cause diarrheal disease in mammals and birds^17^. Three novel astroviruses were identified in *Rattus tanezumi* from different locations (Danzhou, Chengmai, and Baoting). All three strains—Hainan rodent astrovirus 1, Hainan rodent astrovirus 2, and Hainan rodent astrovirus 3—exhibited consistently low aa identities (ranging from 71.5% to 71.7%) to Wufeng rodent astrovirus 1.

*Arteriviridae* is a family of enveloped, positive-sense RNA viruses currently comprising six subfamilies and thirteen genera^18,19^. Multiple novel arteriviruses were identified in *Rattus norvegicus* across different sampling locations. Two strains (Hainan rodent arterivirus 1 and Hainan rodent arterivirus 2) were identified in Dingan, showing 69.77% and 69.43% aa identity, respectively, to arteriviruses from *Rattus exulans*. Seven additional strains were identified in Haikou, exhibiting sequence identities ranging from 67.21% to 78.42% to reference arteriviruses. Among these, four strains (Hainan rodent arterivirus 3, Hainan rodent arterivirus 4, Hainan rodent arterivirus 5, and Hainan rodent arterivirus 6) showed closest aa identity to Norway rat arterivirus with 73.65–73.96% aa identity, while three strains Hainan rodent arterivirus 7, Hainan rodent arterivirus 8, and Hainan rodent arterivirus 9) were most similar to Arteriviruses from *Rattus exulans* with 67.21–78.42% aa identity. Phylogenetic analysis reveals these novel sequences form distinct clusters within the *Arteriviridae* family, suggesting both host-adapted evolutionary patterns and genetic evidence compatible with possible viral exchange among rodent species.

*Picornaviridae* are non-enveloped, positive-sense, single-stranded RNA viruses with non-segmented genomes^20^. This study identified 50 novel viruses with complete genomes across diverse rodent hosts, with the highest viral diversity observed in *Rattus norvegicus* (18) and *Rattus tanezumi* (23), which collectively harbored 82% of all identified viruses. These viruses showed varying degrees of aa identity to known viruses, with Hainan rosavirus-like viruses (10) exhibiting 75.89-78.02% aa identity to Rosavirus C, primarily identified in *Rattus norvegicus* and *Rattus tanezumi*. Hainan rabovirus-like viruses (6) demonstrated 73.55–79.15% aa identity to Rabovirus A across multiple host species, while Hainan rodent kobuviruses (4) showed particularly high aa identity (73.99–74.07%) to Aichivirus A8, exclusively in *Rattus norvegicus*. Both *Rattus norvegicus* and *Rattus tanezumi* showed similar patterns of viral types, hosting *Rosavirus*, *Rabovirus*, and *Kobuvirus*, while other rodent species exhibited more limited but distinct viral profiles.

*Picobirnaviridae* are non-enveloped, double-stranded RNA viruses with segmented genomes^21,22^. This study revealed 58 novel picobirnaviruses with complete genomes across diverse rodent hosts, exhibiting distinct host-specific distribution patterns. *Rattus tanezumi* (23) predominantly carried viruses showing 67.5–74.8% aa identity to Marmot picobirnavirus and 67.1–77.0% aa identity to Porcine picobirnavirus. *Rattus andamanensis* (8) primarily hosted viruses with 65.0–78.2% aa identity to Rat picobirnavirus and 66.6–77.6% aa identity to Porcine picobirnavirus. *Niviventer huang* (5) exhibited viruses demonstrating 66.4–78.2% aa identity to Rat picobirnavirus and 66.1% aa identity to picobirnavirus associated *Rattus norvegicus*.

### Recombination analysis of eight novel viruses

We analyzed recombination sites in the genomes of the eight novel viruses from the *Astroviridae* (2), *Dicistroviridae* (3), *Picobirnaviridae* (2), and *Picornaviridae* (1) families^23^, seven of which were derived from *Rattus tanezumi* and *Rattus norvegicus*.

**Fig. 5:**
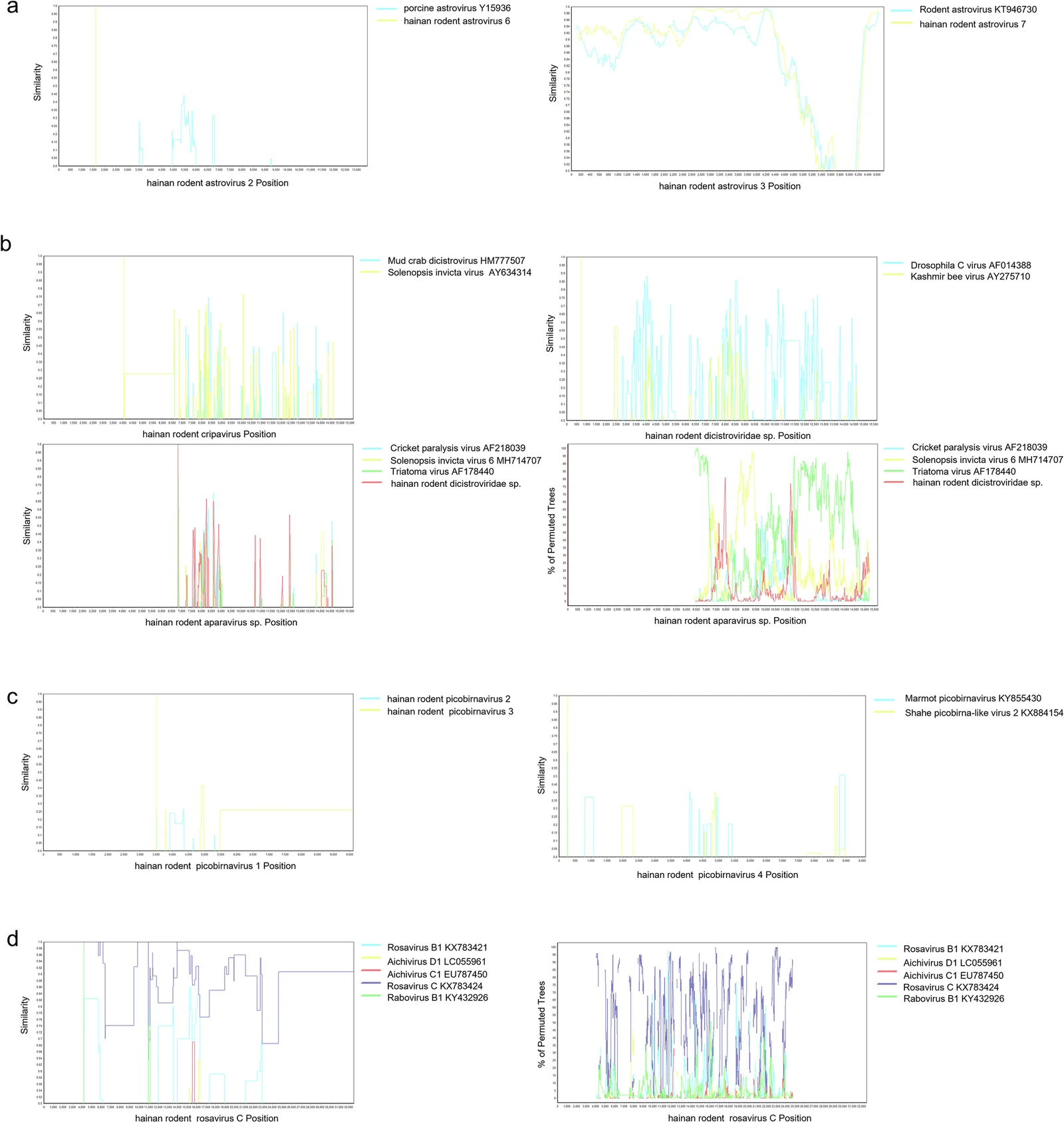
Recombination analysis of eight novel viruses identified in this study. **a** BootScan and similarity plots for *Astroviridae* (Hainan rodent astrovirus 2/3), showing pronounced similarity declines in Hainan rodent astrovirus 2 and Hainan rodent astrovirus 3 with BootScan support. **b** BootScan and similarity plots for *Dicistroviridae* (Hainan rodent cripavirus, Hainan rodent dicistroviridae sp. and Hainan rodent aparavirus sp.). **c** BootScan and similarity plots for *Picobirnaviridae* (Hainan rodent picobirnavirus 1/4). **d** BootScan and similarity plots for *Picornaviridae* (Hainan rodent rosavirus C). All plots use similarity (y-axis) and genomic position (x-axis) to illustrate recombination events, with reference viruses labeled by color.

Several recombination events were identified within *Astroviridae* family. In Hainan rodent astrovirus 2, two intervals (3521–3601 nt and 4961–5981 nt) showed pronounced similarity declines (0.1–0.4). A comparable signal was identified in Hainan rodent astrovirus 3 between 2600 and 3000 nt, with similarly low BootScan support (<50%) (Fig. 5a). *Dicistroviridae* family displayed more extensive and complex recombination patterns. The genome of Hainan rodent cripavirus contained multiple recombinant blocks with sharply shifted similarity and BootScan support (e.g., 7720–8000 nt, 40–60%; 11200–11340 nt, 77%). Similar events were identified in Hainan rodent dicistroviridae sp. and Hainan rodent aparavirus sp. within the 4000–4500 nt and 7000–8000 nt intervals (Fig. 5b). *Picobirnaviridae* and *Picornaviridae* families exhibited recombination characterized by abrupt transitions. Hainan rodent picobirnavirus 1 showed a sharp drop in similarity (<0.3) after position 1500 nt, Hainan rodent picobirnavirus 4 also showed pronounced variation over a 1000-nt span (Fig. 5c). Hainan rodent rosavirus C displayed oscillating similarity (0.4–0.9) and support rates (20–80%) across a 2000-nt region, indicating multiple breakpoints (Fig. 5d).

### Distribution of human pathogenic viruses

**Fig. 6:**
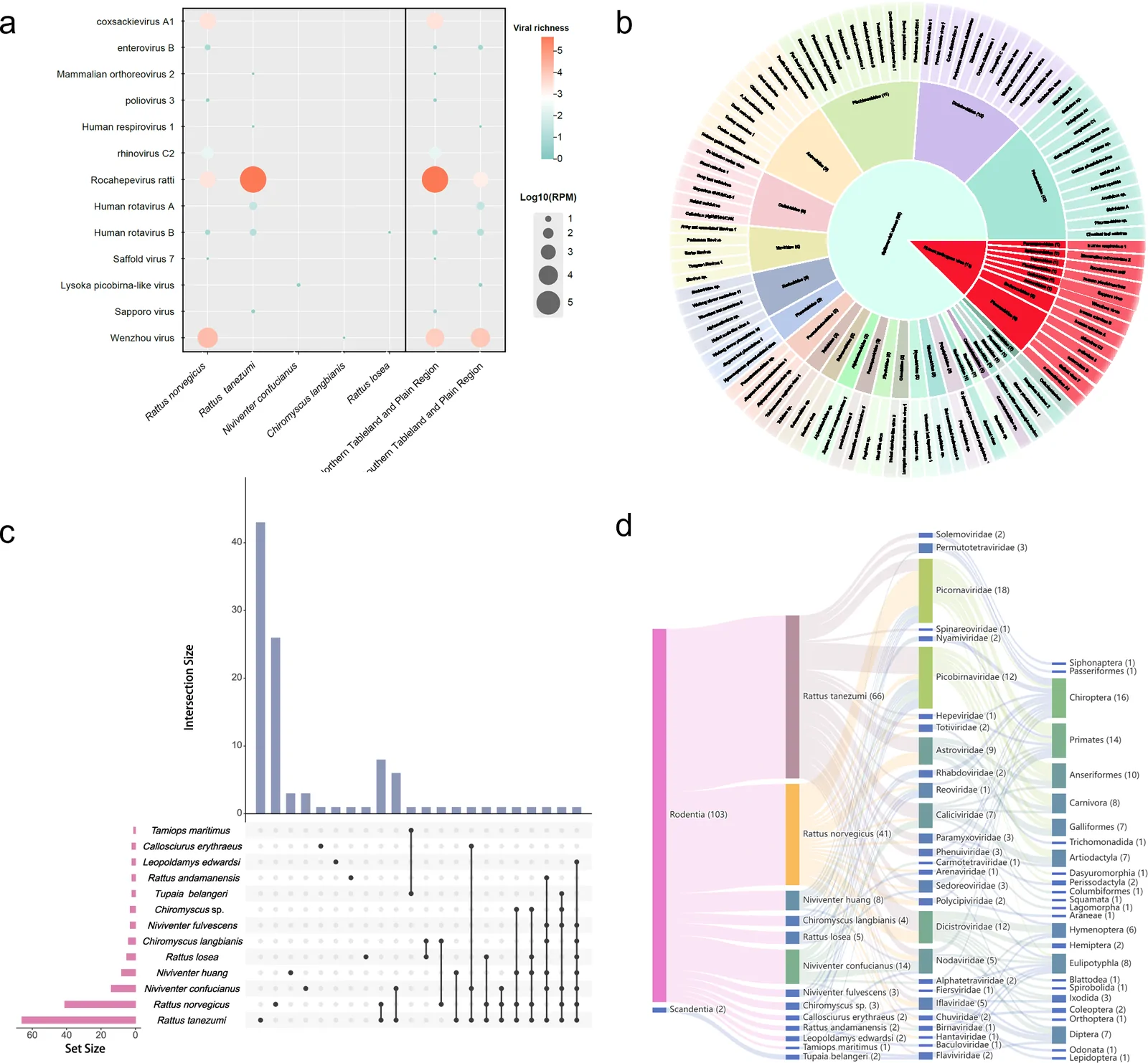
Distribution, sharing patterns, taxonomic profiles, and cross-species transmission networks of zoonotic and cross-species viruses in small mammals and sampling regions. **a** Distribution of human pathogenic viruses across five rodent species and two sampling regions (Northern and Southern), where color intensity denotes viral richness, and circle size denotes Log10(RPM). **b** Sunburst plot illustrating the taxonomic hierarchy of 105 identified cross-species transmissible viruses (encompassing 28 viral families). Among these cross-species viruses, 13 are confirmed human pathogens—highlighted by red-filled sectors—while an additional 92 exhibit spillover potential; viruses from different families are distinguished by distinct color fillings. **c** Venn diagram illustrating the counts of unique and shared cross-species transmissible viruses among 13 small mammal species included in this study. **d** Sankey diagram visualizing cross-species transmission networks of the 28 viral families across 26 host orders, with nodes color-coded to distinguish host orders and viral families, and node border colors highlighting viral families capable of transmitting among multiple host orders.

We identified 13 human pathogenic viruses across five rodent species (*Rattus norvegicus*, *Rattus tanezumi*, *Niviventer confucianus*, *Chiromyscus langbianis*, and *Rattus losea*) and two sampling regions (Northern and Southern), spanning eight viral families (Fig. 6a). These viruses included representatives from *Picornaviridae* (5, coxsackievirus A1, enterovirus B, poliovirus 3, rhinovirus C2, saffold virus 7), *Sedoreoviridae* (2, Human rotavirus A, Human rotavirus B), *Arenaviridae* (1, Wenzhou virus), *Paramyxoviridae* (1, Human respirovirus 1), *Caliciviridae* (1, Sapporo virus), *Hepeviridae* (1, Rocahepevirus ratti), *Picobirnaviridae* (1, Lysoka picobirna-like virus), and *Spinareoviridae* (1, Mammalian orthoreovirus 2). The majority (9/13) of these viruses were enteric viruses.

To comprehensively elucidate the distribution patterns of human pathogenic viruses across rodent species and the north-south sampling regions, we performed a virus abundance analysis. At the species level, our results demonstrated that the majority of human pathogenic viruses (12/13) were identified in *Rattus norvegicus* (8/13) and *Rattus tanezumi* (6/13), with notably high viral abundance of Rocahepevirus ratti observed in both species. Additionally, *Rattus norvegicus* harbored relatively high abundances of coxsackievirus A1 and Wenzhou virus, which were not identified in *Rattus tanezumi*. At the regional level, the Northern region harbored a greater number of human pathogenic viruses (10/13) compared to the Southern region (7/13). Both regions exhibited relatively high abundances of Rocahepevirus ratti and Wenzhou virus. Notably, six human pathogenic viruses were exclusively identified in the Northern region, including coxsackievirus A1, Mammalian orthoreovirus 2, rhinovirus C2, poliovirus 3, Saffold virus 7, and Sapporo virus, with coxsackievirus A1 being particularly abundant. In contrast, only two viruses, Human respirovirus 1 and Lysoka picobirna-like virus, were uniquely identified in the Southern region.

### Analysis of cross-species transmission viruses

Based on an integrative analysis of our sequencing data and documented virus records from the NCBI database, a total of 105 known virus species (19.9%, 105/527) were identified as capable of cross-species transmission. These viruses were identified in two or more vertebrate and invertebrate host orders and span 28 viral families, with the most prominent being *Picornaviridae* (18), *Dicistroviridae* (12), *Picobirnaviridae* (12), *Astroviridae* (9), and *Caliciviridae* (7) (Supplementary Table 8 and Fig. 6b). At the species level, diversity of cross-species viruses varied markedly among hosts, ranging from a single virus in *Tamiops maritimus* to 66 in *Rattus tanezumi* and 41 in *Rattus norvegicus*—which together accounted for 88.6% (93/105) of the cross-species viruses (Supplementary Table 6 and Supplementary Fig. 3). Importantly, a core set of 14 cross-species viruses were consistently identified in both *Rattus tanezumi* and *Rattus norvegicus* (Fig. 6c), underscoring their role as major hosts and potential mediators of viral sharing.

To further elucidate patterns of cross-species virus transmission at the host order level, we constructed a host-virus network incorporating 105 viruses and 26 host orders spanning both vertebrates and invertebrates (Fig. 6d). The viruses were widely distributed among diverse host groups, with the principal reservoirs including Chiroptera (16), Primates (14), Anseriformes (10), as well as Carnivora (8), Eulipotyphla (8), Diptera (7), Artiodactyla (7), Galliformes (7), and Hymenoptera (6). We observed that cross-species transmission events occurred most frequently between rodents and bats (Chiroptera), likely attributable to their broad natural distribution. Similarly, primates ranked second in cross-species viral overlap with rodents (14), a pattern potentially driven by high ecological niche overlap that increases opportunities for cross-species exposure. These findings highlight the important role of rodents (especially *Rattus tanezumi* and *Rattus norvegicus*) as potential hubs in viral correlation networks, effectively bridging transmission between distant hosts—such as humans and bats.

## Discussion

The ecological distribution and evolutionary dynamics of EIDs within animal reservoirs constitute a fundamental driver of zoonotic disease emergence^16^. Within this context, rodents are recognized as important viral carriers^24^, yet the specific species acting as key hosts—particularly in high-risk regions like Hainan Island, which exhibits both high biodiversity and rapid anthropogenic disturbance^3^—remain poorly defined. To address this systematically, our study constructed a gut viromic map of 16 small mammals covering 18 cities and counties across Hainan Island (excluding Sansha), surpassing the majority of previous studies in both spatial and analytical depth^3–5,10,12,17^, and thereby shedding initial light on this “black box.” Our results revealed 527 RNA viral species, along with 175 complete and nearly complete viral genomes, substantially enriching the known viral repertoire. Further phylogenetic and host-association analyses identified *Rattus norvegicus* and *Rattus tanezumi* as key nodes within the network of viral evolution and transmission. Our work thus not only clarifies, for the first time, the central role of *Rattus norvegicus* and *Rattus tanezumi* but also reveals that the viral diversity in each of these species surpasses the previous per-species maximum of 141 viruses reported in virome studies^25^. These results not only offer a scientific basis for targeted early warning and intervention against zoonotic threats but also advance pathogen surveillance in Hainan—an international hub—from a state of “unknown” toward one of “informed awareness”.

This study revealed that host taxonomy plays a primary role and geographical location plays a secondary role in determining gut viral diversity—a finding consistent with several previous studies in viral ecology^26,27^. Furthermore, this is further supported by the result that several additional metadata variables (e.g., season and altitude) showed no significant association with the virome structure. This outcome likely stems from the ecological homogeneity across Hainan Island, where temperature, humidity, elevation, and other factors exhibit minimal variation, creating a relatively uniform ecological backdrop for small mammal hosts. However, unlike earlier research that reported no significant difference in viral diversity between *Rattus* and other hosts (such as *Mus, Niviventer*, and *Meriones*)^28^, our findings suggest *Rattus norvegicus* and *Rattus tanezumi* as important hubs of viral diversity within the Hainan Island ecosystem. Specifically, these two species harbored 83.9% of the total viruses identified, providing compelling confirmation of their role as “viral convergence hotspots” in the local ecosystem. Such a distribution pattern may stem from a combination of intrinsic physiological traits and extrinsic ecological factors^23^. From a physiological perspective, rat may act as highly efficient reservoir hosts, potentially due to co-evolved traits—including possible immune adaptations^29^—that enable them to tolerate a broad range of viruses without displaying clinical disease. Ecologically, their broad habitat adaptability^5^, high population density^7^ are likely to establish a stable ecological setting that facilitates viral proliferation and persistence among hosts. This setting may promote the concurrent accumulation of multiple viral lineages, which could contribute to the observed high viral diversity in these two species. Furthermore, while prior comparative data suggested lower viral diversity in Hainan than in most other provinces^3^, our in-depth analysis revealed that the island’s tropical ecological conditions actually support a highly rich viral community. Notably, both viral diversity and richness in northern Hainan were markedly higher than in the south, suggesting that surveillance efforts should prioritize such high-viral-density regions. Critically, we redirect attention from wildlife to human-commensal species, especially *Rattus norvegicus* and *Rattus tanezumi*, which carry high spillover viral loads at human-wildlife interfaces. These insights offer a new foundation for monitoring and preventing zoonotic diseases.

Phylogenetic analysis further reveals that novel RNA viruses in Hainan’s small mammals exhibit clear host-specific evolutionary clustering, indicating that viral evolution in this ecosystem is substantially constrained by host phylogeny. This pattern is most evident in *Rattus tanezumi* and *Rattus norvegicus*, which harbored 23 and 49 novel viruses, respectively, collectively accounting for 68.6% (72/105) of all newly identified viruses. This substantial uncharacterized diversity reinforces their ecological role as pivotal “viral innovation hosts.” The prolific viral evolution observed in these two hosts likely stems from a combination of their physiological and ecological traits^30^. The frequent co-occurrence of *Rattus tanezumi* and *Rattus norvegicus* with diverse mammalian, avian, and invertebrate species sustain ecological overlap that facilitates host switching—promoting the emergence and accumulation of novel RNA viruses. The long-term coexistence of numerous viruses within a single host provides idealized conditions for genetic recombination and mutation.

Notably, our screening identified diverse RNA viruses—including members of *Permutotetraviridae*, *Dicistroviridae*, *Chuviridae*, and *Phenuiviridae*—in rodent gut samples, thereby extending the known taxonomic range of these groups to rodents in southern China. *Permutotetraviridae* were historically described in invertebrates but have recently been reported in bats^4^; *Dicistroviridae* are characterized by a dicistronic genome structure and are primarily associated with insects^23^. *Chuviridae* exhibit remarkable genomic diversity, encompassing circular, linear, segmented^14^, and unsegmented negative-sense RNA forms^15^; and *Phenuiviridae* include pathogens of public health concern such as SFTSV^16^. However, as all sequences were recovered from digestive tract samples, the observed viral signals may reflect either active infection or recent dietary intake of infected arthropods or other organisms. Without evidence from non-digestive tissues or serological data, we cannot infer replication competence or reservoir status for these viruses. Nevertheless, the identification of such viruses across multiple taxonomic orders suggests that transmission was likely facilitated by shared habitats^16^. The inherent genomic plasticity of RNA viruses enables their adaptation to new mammalian hosts. In summary, within the complex viral evolutionary landscape of Hainan, *Rattus tanezumi* and *Rattus norvegicus* may function as potential hubs, playing a critical and disproportionate role in the emergence and persistence of viral diversity.

*Rattus norvegicus* and *Rattus tanezumi* have long been recognized as significant reservoirs of zoonotic viruses, such as hantaviruses, coronaviruses, and hepatitis E virus. This study substantially reinforces their status as primary reservoir hosts through viral spectrum analysis, revealing that among the thirteen identified human pathogenic viruses, *Rattus norvegicus* and *Rattus tanezumi* collectively harbored twelve of them, thereby demonstrating a distinct pattern of host aggregation. Geographically, the greater diversity of human pathogenic viruses in the northern region (10/13) likely reflects ecological gradients shaped by increased anthropogenic pressure^2,4^. Among the 105 viruses with cross-species transmission potential, *Rattus norvegicus* and *Rattus tanezumi* collectively harbored 88.6% (93/105), acting as central nodes within the structured networks of multi-host virus sharing. This finding strongly aligns with and substantially extends previous regional and global reports^24,31^. These findings highlight the necessity of prioritizing targeted surveillance of synanthropic *Rattus norvegicus* and *Rattus tanezumi* in northern Hainan as a strategic step to enhance pandemic preparedness.

Our broad-based metatranscriptomic surveillance across diverse mammalian hosts revealed extensive cryptic circulation of cross-species viruses, with *Rattus norvegicus* and *Rattus tanezumi* appearing as prominent nodes in host–virus transmission networks within the studied ecosystems. This underscores the value of integrating unbiased viral discovery with network-informed risk assessment to identify locally relevant reservoirs. Future efforts should prioritize experimental characterization of high-spillover-risk viruses and longitudinally track integrated human–animal serosurveys to detect silent transmission chains^32^, enabling evidence-based interventions that are both targeted and grounded in comprehensive surveillance.

## Methods

### Study site and animal sampling

From June 2023 to December 2024, to investigate the virome characteristics of small mammal species on a tropical island, we conducted sampling across 18 cities and counties of Hainan Island (excluding Sansha; Supplementary Table 1), with each location sampled only once during this period. The animals were captured using folding metal traps baited with Youtiao (occasional peanut supplement)^33^. Among these, five species—*Callosciurus erythraeus*, *Dremomys rufigenis*, *Neohylomys hainanensis*, *Tamiops maritimus*, and *Tupaia belangeri*—all listed as “Terrestrial Wildlife with Important Ecological, Scientific, and Social Value”^34^ were not captured alive but were obtained from animals that had already died. All animal procedures, including trapping and sampling, were approved by the Ethics Committee of the National Institute of Infectious Diseases Control and Prevention, Chinese Center for Disease Control and Prevention (No. ICDC-2023-023, ICDC-2024-036).

Following anesthesia, captured animals were euthanized by cervical dislocation. Seven organs—brain, gut, kidney, liver, lung, spleen, and heart—were collected, immediately preserved in RNAlater, and stored at –80 °C. Molecular species identification was performed using nucleic acids extracted from individual liver samples, via *cytochrome b* (*cytb*) gene amplification and Sanger sequencing (Supplementary Table 1)^23^. Based on these confirmed species identities and sampling locations (city/county), we applied a stratified random sampling approach derived from standard cross-sectional survey methodology^8^. Using the conventional sample size formula for prevalence estimation—with a 95% confidence level, 5% margin of error, and a conservative prevalence assumption of *P* = 0.5—the maximum required sample size per species–location stratum was calculated as 21 individuals. For strata exceeding this threshold, 21 individuals were randomly selected; all others were retained in full. This procedure yielded a total of 1284 individuals designated for virome analysis (Supplementary Table 1). For these individuals, nucleic acids were separately extracted from gut samples, and 1 μg of each extract was pooled based on host species and sampling location, ultimately forming 125 pools (1–21) for subsequent metatranscriptomic analysis. At each sampling city and county, we recorded geographic coordinates, elevation, and microenvironmental parameters (temperature and relative humidity) (Supplementary Table 1).

### RNA extraction, library preparation, and sequencing

Total RNA was isolated from gut samples using the AllPrep PowerViral DNA/RNA Kit (QIAGEN, Germany) following the manufacturer’s protocol, and subsequently diluted to a final concentration of 50 ng/μL for downstream applications^35^. To minimize ribosomal RNA (rRNA) contamination, depletion was performed using the Ribo-MagOff rRNA Depletion Kit (Vazyme, China)^24^, which employs magnetic bead-based sequence-specific capture technology. For cDNA library preparation, the VAHTS Universal V10 RNA-seq Library Prep Kit for Illumina (Vazyme, China) was utilized following a standardized workflow: RNA fragmentation was carried out first, followed by reverse transcription, terminal adenylation, and adapter ligation^2^. The resulting cDNA libraries were purified using VAHTS RNA Clean Beads (Vazyme, China) and amplified via PCR^36^. Library concentration was determined using the Qubit dsDNA BR Assay (Thermo Fisher Scientific, USA)^10^. A total of 125 pools were constructed based on species and sampling cities/counties through equimolar mixing (Supplementary Table 2). All libraries subsequently underwent 150-bp paired-end sequencing on the NovaSeq X Plus platform (Illumina, USA)^37^.

### Sequence analysis and viral species demarcation

Raw sequencing reads were processed using FastQC (v0.11.5) for quality assessment, followed by filtration to remove low-quality bases. rRNA contamination was identified through sequence alignment against the SILVA rRNA database using Bowtie (v2.5.2)^3^, and matching reads were excluded from subsequent analyses. The filtered high-quality reads underwent de novo assembly using SPAdes (v4.2.0)^38^, which reconstructed contiguous sequences (contigs) from overlapping fragments. Assembled contigs were annotated by comparing their predicted protein sequences to the GenBank non-redundant protein database through DIAMOND BLASTx (2.1.8)^16^, retaining only significant matches for further analysis^35^. Contigs showing amino acid (aa) identity to viral proteins were classified based on taxonomic assignments: those aligning to *Riboviria*-associated entries (NCBI Taxonomy ID: 2559587) were designated as candidate viral sequences^6,36^. Assembly quality was assessed by mapping cleaned reads back to viral contigs using Bowtie2 (v2.4.5), with alignments processed by SAMtools (v1.5) for sorting and indexing^1^.

For the taxonomic identification of viruses and the identification of new viruses, we first performed BLASTx against the NCBI NR database (May 2024 release; taxonomy ID: 2559587)^16^. For each contig, we retrieved all significantly matching viral sequences and supplemented them with representative members of the corresponding viral family to construct phylogenetic trees based on RdRp domains. Viral taxonomy was assigned following the ICTV13 species demarcation criteria where available^2^. For viral groups lacking formal ICTV guidelines, contigs exhibiting ≤80% aa identity in the RdRp to the closest reference strain were designated as novel species^39^.

For cross-species transmission analysis of known viral species, the potential host was defined as the hosts documented in ICTV13 reports or the NR database (May 2024 release) for the phylogenetically closest reference virus. Cross-species viruses were defined as viral species that were identified in two or more host orders^10^. This definition was based on a comprehensive assessment that combined our metatranscriptomic assemblies and publicly available virus–host records from the NCBI database (May 2024 release)^16^. For network construction, we generated a bipartite association matrix that linked each cross-species virus to all the host orders in which it was identified. Subsequently, the network was visualized as a Sankey diagram using the networkD3 package (v2.0.0) in R (v4.2.2). Additionally, we summarized and illustrated the distribution and overlap of cross-species viruses among mammalian hosts using Venn diagrams generated with the VennDiagram package (v1.7.3) in R (v4.2.2).

### Phylogenetic analysis

Multiple sequence alignments were constructed for conserved RdRp domains using MAFFT (v7.490)^4^, followed by removal of poorly aligned regions with TrimAl (v1.4) using the automated1 heuristic^25^. Phylogenetic trees were inferred using the maximum likelihood (ML) method as implemented in FastTree (v2.1.11) with 1,000 bootstrap replicates to assess branch support^7^. Final tree visualization and annotation were carried out in R (v 4.2.2) using the ggtree package^33^.

### Recombination analysis

Recombination analysis was conducted using SimPlot (v3.5.13)^8^. Sequence homology among selected viruses was assessed via SimPlot’s basic analysis and visualized as network diagrams. Bootscanning used a 200-nt sliding window (20-nt steps) with Kimura 2-parameter model and Neighbor-Joining method (1000 bootstrap replicates)^5^.

### Statistical analysis

To evaluate the impacts of host species and sampling region on viral communities, we conducted a comprehensive analysis of both alpha and beta diversity using the vegan (v2.6.4) and stats packages in R (v4.2.2)^40^. Beta diversity was assessed via Bray–Curtis dissimilarity, and permutational multivariate analysis of variance was performed using the adonis2 function in vegan to test the effects of the 16 host species and the northern versus southern regions of Hainan Island on viral community composition^41^. For alpha diversity, quantified by the Shannon index and observed richness, pairwise comparisons between the two regions and among host species were conducted using t-tests from the stats package, with *P*-values adjusted for multiple testing using the Benjamini–Hochberg procedure^42^. Visualization of diversity metrics and ordination results was generated with ggplot2 (v3.4.0).

## Supplementary information

Supplementary information

Supplementary Tables 1–8

## Data Availability

Data is deposited in the National Microbiology Data Center (NMDC) with accession numbers NMDCN00091GU (https://nmdc.cn/resource/genomics/sequence/detail/NMDCN00091GU).
