# Supplementary material for: Rattus norvegicus and Rattus tanezumi as hub hosts for viral diversity and potential spillover on Hainan Island, China
Source: NPJ Biofilms Microbiomes. 2026 Jun 22;12:196. doi: 10.1038/s41522-026-01056-x (PMC13649783; doi:10.1038/s41522-026-01056-x)
Supplement: Supplementary file 1 [file 41522_2026_1056_MOESM1_ESM.pdf]

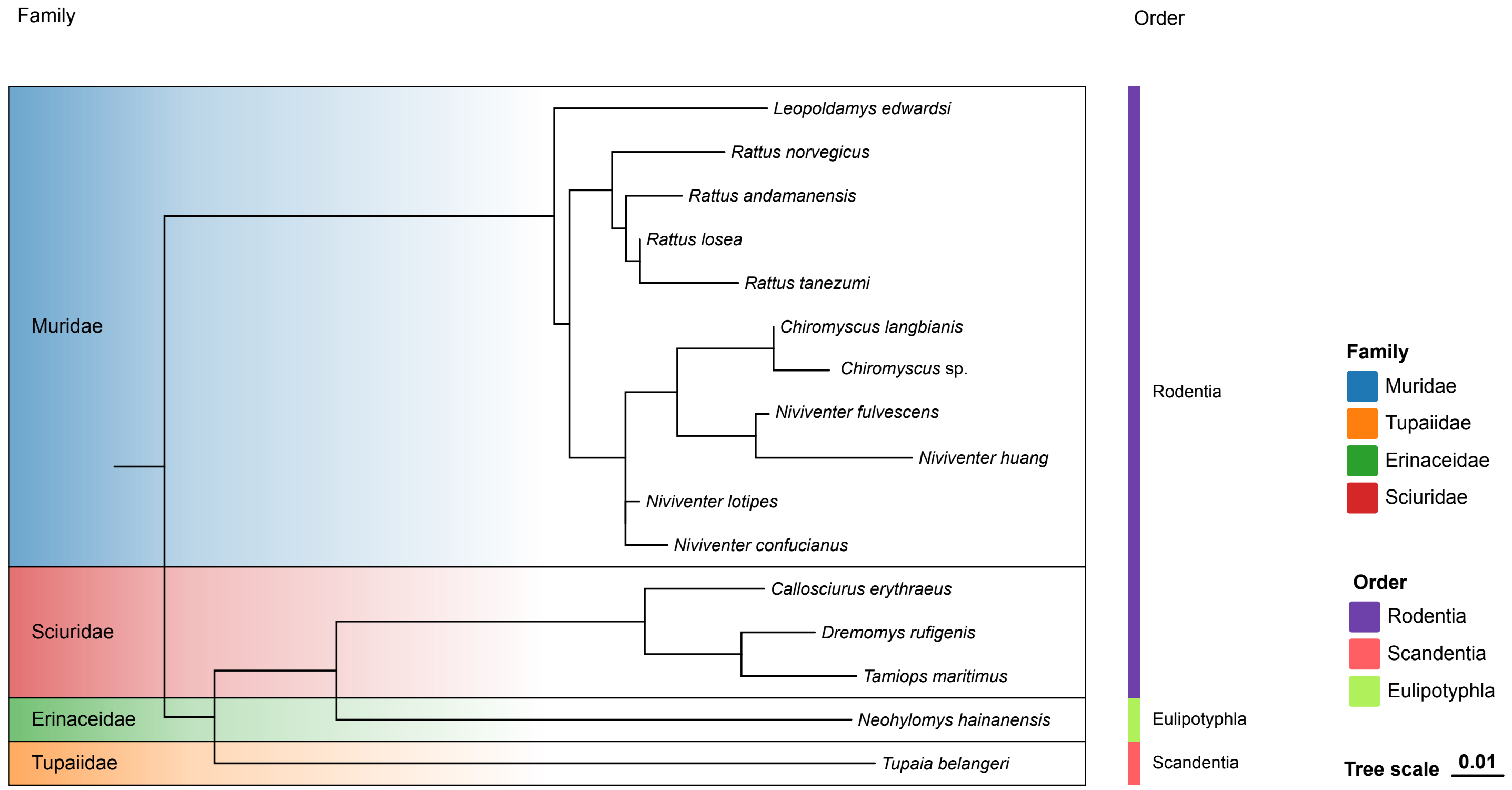

Supplementary Figure 1 Phylogenetic relationships among the 16 small mammal species analyzed in this study: *Rattus tanezumi*, *Rattus norvegicus*, *Niviventer huang*, *Niviventer confucianus*, *Niviventer fulvescens*, *Rattus andamanensis*, *Chiromyscus langbianis*, *Rattus losea*, *Leopoldamys edwardsi*, *Chiromyscus sp.*, *Niviventer lotipes*, *Callosciurus erythraeus*, *Dremomys rufigenis*, *Tamiops maritimus*, *Neohylomys hainanensis*, and *Tupaia belangeri*. Branch lengths are proportional to genetic distance as indicated by the scale bar.

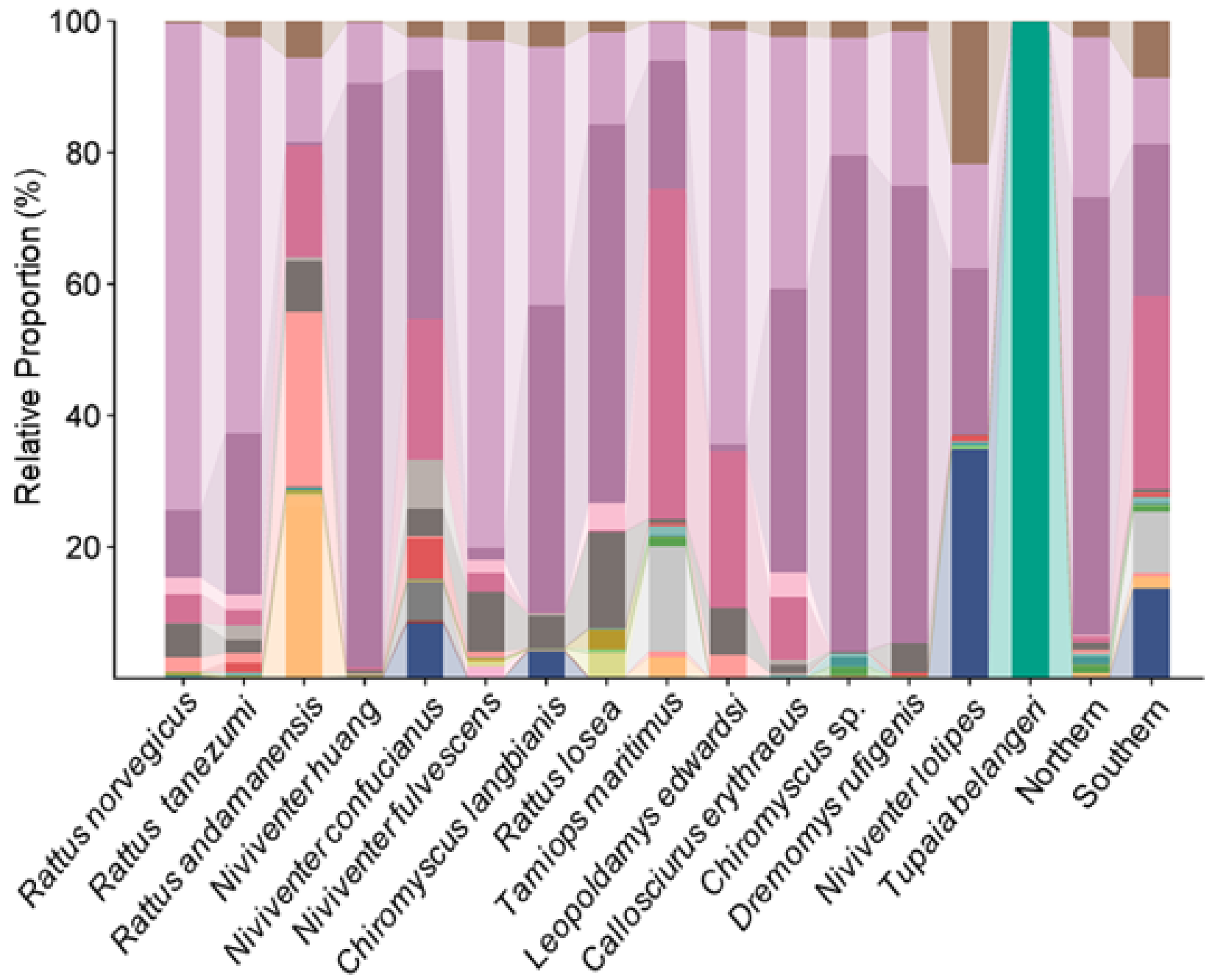

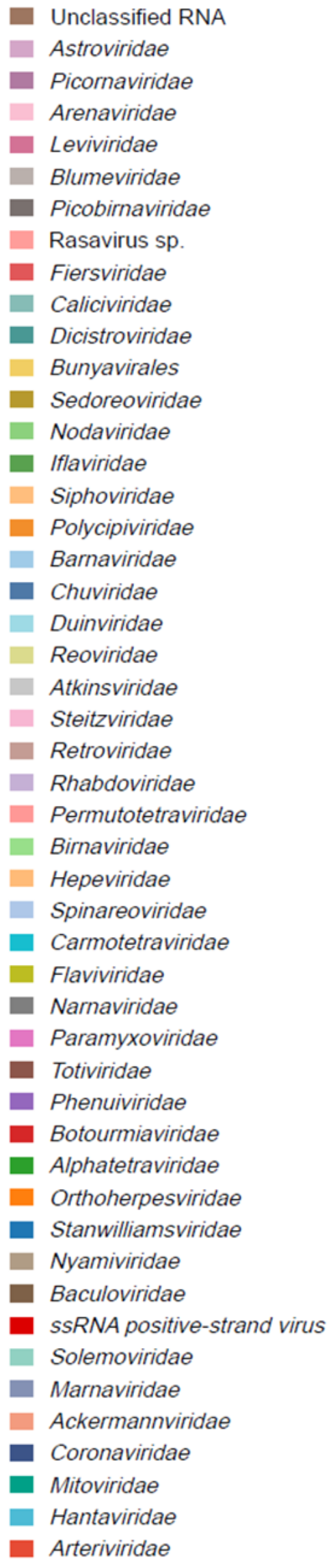

Supplementary Figure 2 The relative abundance of viruses in each library was calculated and normalized as reads per million total reads (RPM). Colors in the heatmap indicate both the small mammal species and the northern vs southern regions of the sampling locations.

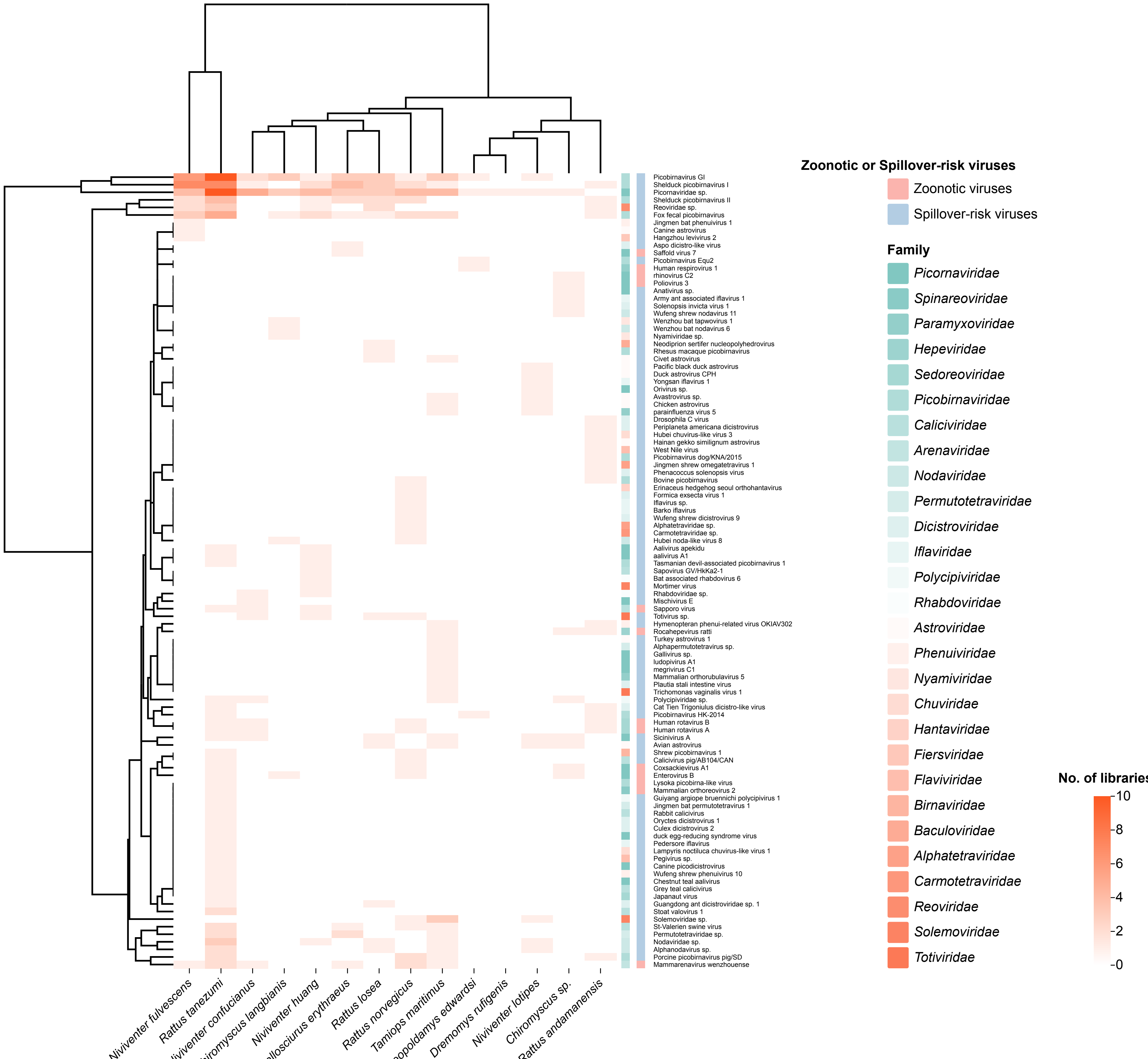

Supplementary Figure 3 Distribution of 105 zoonotic and spillover-risk viruses across 14 small mammal species tested in this study: *Rattus tanezumi*, *Rattus norvegicus*, *Niviventer fulvescens*, *Rattus losea*, *Callosciurus erythraeus*, *Chiromyscus langbianis*, *Niviventer huang*, *Niviventer confucianus*, *Tamiops maritimus*, *Chiromyscus sp.*, *Niviventer lotipes*, *Rattus andamanensis*, *Dremomys rufigenis*, and *Leopoldamys edwardsi*. The red color indicates the number of sequencing libraries in which zoonotic or spillover-risk viruses were identified.
